# Daratumumab improved severe neutrophilia and paraneoplastic bone marrow fibrosis in granulocyte‐colony stimulating factor‐producing multiple myeloma

**DOI:** 10.1002/jha2.901

**Published:** 2024-05-08

**Authors:** Miki Sakamoto, Kohei Shiroshita, Shinya Fujita, Himari Kudo, Ryohei Abe, Sumiko Kohashi, Yuka Shiozawa, Kuniaki Nakanishi, Takaaki Toyama

**Affiliations:** ^1^ Department of Hematology Federation of National Public Service Personnel Mutual Aid Associations Tachikawa Hospital Tokyo Japan; ^2^ Division of Hematology, Department of Medicine Keio University School of Medicine Tokyo Japan; ^3^ Department of Pathology Federation of National Public Service Personnel Mutual Aid Associations Tachikawa Hospital Tokyo Japan

1

In cases of unexplained neutrophilia, granulocyte‐colony stimulating factor (G‐CSF)‐producing tumours should be considered. Although such tumours are mainly reported in solid cancers, a few cases of G‐CSF‐producing multiple myeloma (MM) have recently been reported [1, 2, 3, 4]. Severe neutrophilia raises the possibility of chronic neutrophilic leukaemia (CNL), and the detection of a colony‐stimulating factor 3 receptor (*CSF3R*) mutation is useful in diagnosing CNL [5]. While an association between CNL and plasma cell dyscrasia has been reported [6], a recent study found that plasma cell dyscrasia‐derived G‐CSF could induce CNL‐like neutrophilia without a *CSF3R* mutation [4], suggesting that when clinicians diagnose CNL in a patient with plasma cell dyscrasia, caution should be exercised to avoid misdiagnosis. Although bone marrow (BM) fibrosis (BMF) is frequently observed in myeloproliferative neoplasms (MPNs), MM with BMF has also been reported [7, 8]. However, the number of MM cases with both neutrophilia and BMF is limited; [1, 9] as such, the clinical characteristics and treatment responses remain unclear. Here, we report the second case of G‐CSF‐producing MM complicated with paraneoplastic BMF during disease progression; to our knowledge, there is only one other report of this condition to date [1].

A 63‐year‐old man was admitted to our hospital for evaluation of leucocytosis. His medical history included chronic obstructive pulmonary disease. Blood tests showed that his white blood cell (WBC) count was 19,710 /µL (neutrophils, 85.5%) without anaemia, hypercalcaemia (calcium level, 9.3 mg/dL), renal impairment (blood urine nitrogen level, 11 mg/dL; creatinine level, 0.61 mg/dL), or detectable *BCR::ABL1* transcript. BM examination revealed hypercellular marrow with increased immature myeloid cells (Figure 1A), but no increased blasts or BMF (Figure 1B). Thus, a tentative diagnosis of CNL was established, and the patient was followed without treatment. Two years later, he was admitted to the nephrology department after exhibiting elevated urinary protein levels at a health checkup. Blood tests showed a WBC count of 36,100 /µL (neutrophils, 87.8%), haemoglobin level of 12.6 g/dL, and platelet count of 18.8×10^4^/µL. Immunofixation electrophoresis detected κ‐type Bence–Jones protein and the serum free light chain ratio (rFLC) was increased by 122. BM examination revealed a hypercellular marrow and an increase in CD138‐positive plasma cells to 20% (Figure 1C), although no significant BMF was observed (Figure 1D). Based on these results, the patient was diagnosed with κ‐type Bence–Jones protein‐type MM. Because he could not afford the treatment and chose to wait without treatment. Four years after MM's diagnosis, his condition worsened, with a neutrophil count of 39,400 /µL, a haemoglobin level of 10.4 g/dL, and a platelet count of 10.5×10^4^/µL; these results suggested a gradual progression of neutrophilia, anaemia and thrombocytopaenia. The rFLC was elevated to 142. BM aspiration resulted in a dry tap; however, BM biopsy revealed hypercellular marrow (Figure 1E), increased CD138‐positive plasma cell levels (Figure 1F), and grade F2 BMF (Figure 1G). The plasma cells were positive for G‐CSF staining (Figure 1H) and the serum G‐CSF level was 2730 (normal range: 10.5–57.5) pg/mL. Fluorescence in situ hybridization using a BM biopsy did not reveal *IgH*::*MAF* or *IgH*::*FGFR3*. Positron emission tomography showed increased fluorodeoxyglucose uptake throughout the bone marrow and splenomegaly (Figure 1I). We suspected the coexistence of MPNs but could not identify mutations in *JAK2*, *CALR*, *MPL*, or exons 14 and 17 of *CSF3R*. The final diagnosis was a G‐CSF‐producing MM with BMF. The patient was started on bortezomib‐lenalidomide‐dexamethasone (VRD), which led to a rapid improvement in neutrophil count. However, he achieved only a partial response after five courses of VRD. To gain a better treatment response, his treatment was switched to daratumumab‐carfilzomib‐dexamethasone (DKd), and only one cycle of DKd immediately achieved a stringent complete response. We added five cycles of daratumumab‐lenalidomide‐dexamethasone (DRd), and subsequent BM examination confirmed a normocellular marrow (Figure 1J), reduced plasma cell invasion (Figure 1K), reduced BMF (Figure 1L), and no G‐CSF‐producing plasma cells (Figure 1M). Serum G‐CSF levels decreased by 25.3 pg/mL, with negativity for minimal residual disease (MRD).

**FIGURE 1:**
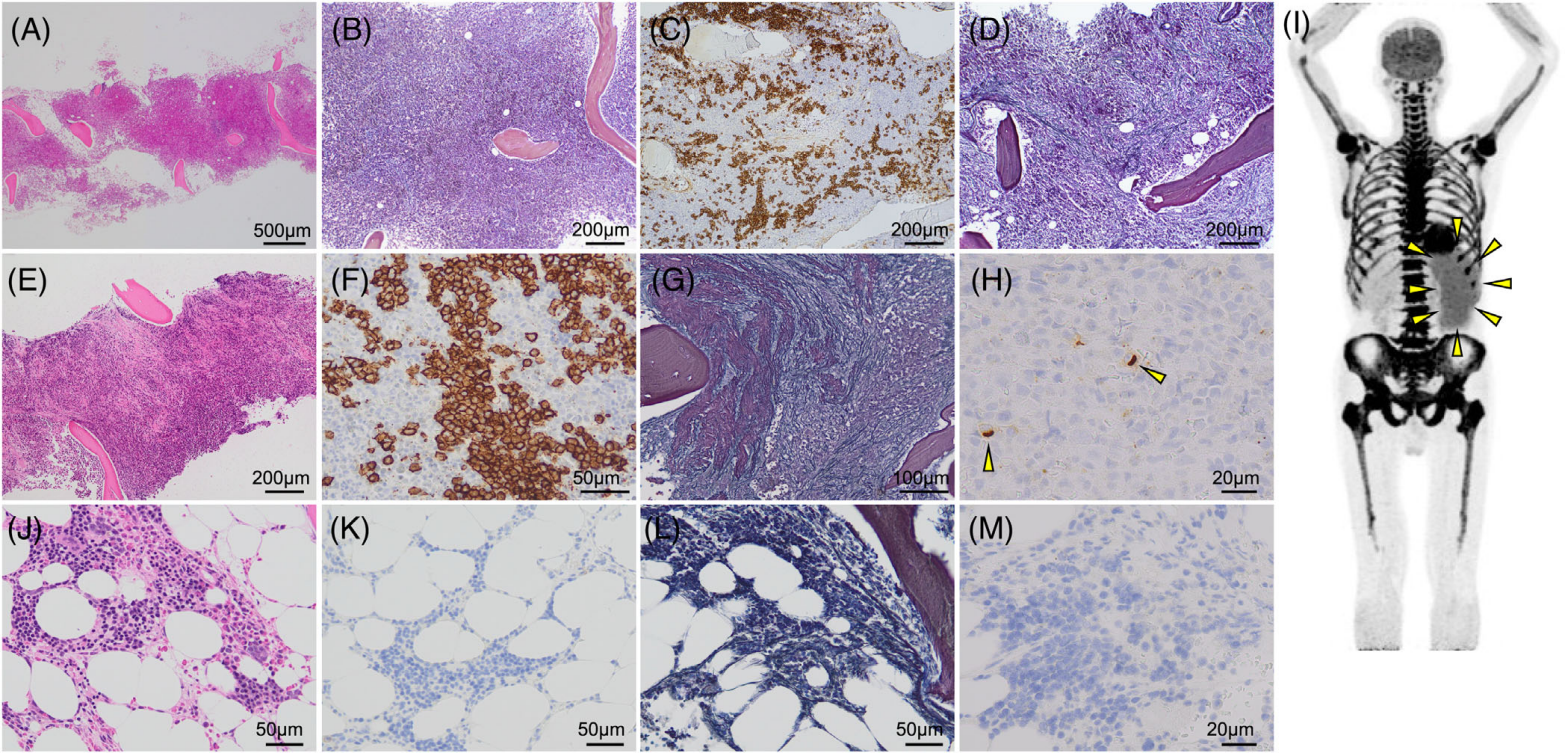
Pathological and imaging results of this patient. (A, B) Bone marrow biopsy results at CNL diagnosis: (A) Haematoxylin‐eosin staining (HE) and (B) silver‐plated staining. (C, D) Bone marrow biopsy results at MM diagnosis: (C) CD138 and (D) silver‐plated staining. (E–H) Bone marrow biopsy results before chemotherapy: (E) HE, (F) CD138, (G) silver‐plated staining and (H) G‐CSF (yellow arrows indicate G‐CSF positive plasma cells). (I) Positron emission tomography scans before chemotherapy revealing fluorodeoxyglucose accumulation in systemic BM and splenomegaly (yellow arrows). (J–M) Bone marrow biopsy results after chemotherapy: (J) HE, (K) CD138, (L) silver‐plated staining and (M) G‐CSF. BM, bone marrow; CNL, chronic neutrophilic leukaemia; G‐CSF, granulocyte‐colony stimulating factor; MM, multiple myeloma.

This case illustrates the challenging diagnosis and treatment course of a rare paraneoplastic BMF in G‐CSF‐producing MM. Cases of MM with neutrophilia and BMF are summarised in Table 1. All patients were ≥60 years old and had myeloma‐defining events. Cases 2 and 3 were negative for MPNs‐related mutations but elevated serum G‐CSF levels were observed. No *CSF3R* mutation, G‐CSF expression of MM cells in immunohistochemical staining of the BM, and the results of monitoring for MRD were confirmed only for our case. In all cases, neutrophilia and BMF improved after chemotherapy.

**TABLE 1:** Case series of multiple myeloma (MM) with neutrophilia and bone marrow (BM) fibrosis.

Case	Age/sex	Neutrophil (/µL)	MDE	M‐protein type	sG‐CSF (pg/mL)	G‐CSF staining in IHC	Genetic mutation	Treatment	Response	Outcome
1 (reference 9)	60F	17,600	Bone	IgA‐λ	NT	NT	NT	VAD	sG‐CSF levels: NT BM fibrosis: improved MRD: NT	unknown
2 (reference 1)	70 M	54,000	Anaemia	IgG‐κ	375	NT	*BCR::ABL1* (‐) *JAK2/MPL/CALR* (‐)	VTD → KCd	sG‐CSF levels: decreased BM fibrosis: improved MRD: NT	Alive
3 (This case)	63 M	39,400	Anaemia	BJP‐κ	2730	Positive	*BCR::ABL1*(‐) *JAK2/MPL/CALR* (‐) *CSF3R* exon 14/17 (‐)	VRd→DKd →VRd→Rd →DRd	sG‐CSF levels: decreased BM fibrosis: improved MRD: negative	Alive

Abbreviations: BM, bone marrow; CSF3R, colony‐stimulating factor 3 receptor; DKd, daratumumab‐carfilzomib‐dexamethasone; DRd, daratumumab‐lenalidemide‐dexamethasone; G‐CSF, granulocyte colony‐stimulating factor; IHC, immunohistochemical staining; KCd, carfilzomib‐cyclophosphamide‐dexamethasone; MDE, myeloma‐defining event; MM, multiple myeloma; MRD; minimal residual disease, Rd, lenalidemide‐dexamethasone; sG‐CSF, serum G‐CSF; NT, not tested; VAD, vincristine‐adriamycin‐dexamethasone; VRd, bortezomib‐lenalidemide‐dexamethasone; VTD, bortezomib‐thalidomide‐dexamethasone.

MM and BMF exhibit distinct clinical characteristics. They are resistant to proteasome inhibitors and immunomodulatory drugs. [8] Indeed, cases 1 and 2 were successfully treated with a cytotoxic agent‐containing regimen but not with the combined treatment of proteasome inhibitors and immunomodulatory drugs. [1, 9] In our case, VRD achieved a partial response only, whereas DKd and DRd enhanced the response to a stringent complete response and negativity for MRD, respectively. Considering that the latter efficiently treats G‐CSF‐producing MM, [2] a daratumumab‐based regimen, as well as a cytotoxic agent regimen, may be good choices for G‐CSF‐producing MM with BMF.

The mechanism of action of BMF in MM remains unclear. Although BMF is a risk factor for extramedullary MM, [7] our patient did not have any extramedullary disease at diagnosis. MPNs‐related mutations were not detected, and BMF recovered after treatment in our and previous cases. [1, 9] These observations suggest an MM‐related paraneoplastic mechanism of BMF. MM cells can produce several cytokines, including interleukin‐6 and tumour growth factor β1 (TGF‐β1). [10] TGF‐β is a profibrotic cytokine and therefore plays an important role in BMF. [11] Based on these findings, one possible hypothesis is that paraneoplastic TGF‐β1 induces BMF in the MM. Future studies should further investigate this aspect.

In summary, we report a case of G‐CSF‐producing MM with BMF that was successfully treated with daratumumab. Unexplained neutrophilia with urinary protein should be considered in G‐CSF‐producing MM, and performing a BM biopsy with G‐CSF staining and checking serum G‐CSF levels are strongly recommended. Heightened awareness and further accumulation of cases are necessary to clarify the pathogenesis, optimal therapy, and mechanism of action of BMF in G‐CSF‐producing MM.

## AUTHOR CONTRIBUTIONS

Kohei Shiroshita and Shinya Fujita designed the study. Miki Sakamoto, Kohei Shiroshita, Shinya Fujita, Himari Kudo, Ryohei Abe, Sumiko Kohashi, Yuka Shiozawa and Takaaki Toyama are physicians of this patient. Miki Sakamoto, Kohei Shiroshita and Shinya Fujita collected the clinical data. Kuniaki Nakanishi performed pathological analyses. Miki Sakamoto, Kohei Shiroshita and Shinya Fujita wrote the manuscript. Kohei Shiroshita, Shinya Fijita, and Takaaki Toyama supervised the manuscript preparation. All authors contributed to drafting the manuscript and approved its submission.

## CONFLICT OF INTEREST STATEMENT

The authors declare no conflict of interest.

## ETHICS STATEMENT

This study does not require the institutional ethics committee.

## CLINICAL TRIAL REGISTRATION

The authors have confirmed clinical trial registration is not needed for this submission.

## PATIENT CONSENT STATEMENT

Informed consent for publication has been obtained from the enrolled patient.

## Data Availability

Not applicable.
